# Unique Regulation of Intestinal Villus Epithelial Cl^−^/HCO_3_^−^ Exchange by Cyclooxygenase Pathway Metabolites of Arachidonic Acid in a Mouse Model of Spontaneous Ileitis

**DOI:** 10.3390/ijms22084171

**Published:** 2021-04-17

**Authors:** M Motiur Rahman, Alip Borthakur, Sheuli Afroz, Subha Arthur, Uma Sundaram

**Affiliations:** Department of Clinical and Translational Sciences, Joan C. Edwards School of Medicine, Marshall University, Huntington, WV 25701, USA; rahmanmd@marshall.edu (M.M.R.); borthakur@marshall.edu (A.B.); afroz@marshall.edu (S.A.); arthursu@marshall.edu (S.A.)

**Keywords:** Cl^−^/HCO_3_^−^ exchange, COX pathway, inflammation, prostaglandins, SAMP1 mice

## Abstract

Electrolytes (NaCl) and fluid malabsorption cause diarrhea in inflammatory bowel disease (IBD). Coupled NaCl absorption, mediated by Na^+^/H^+^ and Cl^−^/HCO_3_^−^ exchanges on the intestinal villus cells brush border membrane (BBM), is inhibited in IBD. Arachidonic acid metabolites (AAMs) formed via cyclooxygenase (COX) or lipoxygenase (LOX) pathways are elevated in IBD. However, their effects on NaCl absorption are not known. We treated SAMP1/YitFc (SAMP1) mice, a model of spontaneous ileitis resembling human IBD, with Arachidonyl Trifluoro Methylketone (ATMK, AAM inhibitor), or with piroxicam or MK-886, to inhibit COX or LOX pathways, respectively. Cl^−^/HCO_3_^−^ exchange, measured as DIDS-sensitive ^36^Cl uptake, was significantly inhibited in villus cells and BBM vesicles of SAMP1 mice compared to AKR/J controls, an effect reversed by ATMK. Piroxicam, but not MK-886, also reversed the inhibition. Kinetic studies showed that inhibition was secondary to altered K_m_ with no effects on V_max_. Whole cell or BBM protein levels of Down-Regulated in Adenoma (SLC26A3) and putative anion transporter-1 (SLC26A6), the two key BBM Cl^−^/HCO_3_^−^ exchangers, were unaltered. Thus, inhibition of villus cell Cl^−^/HCO_3_^−^ exchange by COX pathway AAMs, such as prostaglandins, via reducing the affinity of the exchanger for Cl^−^, and thereby causing NaCl malabsorption, could significantly contribute to IBD-associated diarrhea.

## 1. Introduction

Inflammatory Bowel Disease (IBD) is a chronic, relapsing, and remitting inflammatory disease of the gastrointestinal tract that encompasses both Crohn’s disease (CD) and ulcerative colitis (UC) [1,2,3]. IBD affects more than 3.5 million people in the United States and Europe, with a steep increase in incidence over the past 50 years [4,5]. The etiology of IBD is complex, there is no cure, and the cause of the disease remains unclear, limiting the development of therapeutic strategy to prevent its occurrence. Therefore, the therapy is focused toward mitigation of symptoms, rather than cure of disease [1]. Diarrhea, the most common and disabling morbidity of human IBD, is prevalent in almost 80% of the patients [6]. IBD-associated diarrhea is multifactorial and appears to be the outcome of intricate pathophysiological events arising from widespread and sustained mucosal inflammation [6]. Akin to all diarrheal diseases, decreased absorption, increased secretion, or both, of fluid and electrolyte is a common manifestation of IBD-associated diarrhea [7,8,9]. However, the majority of the studies investigating its pathophysiology have suggested that the predominant mechanism of diarrhea in IBD involves impairment of electroneutral NaCl absorption, rather than altered anion secretion [10]. Electroneutral NaCl absorption in the mammalian small intestine occurs via a coupled mechanism of Na^+^/H^+^ exchange and Cl^−^/HCO_3_^−^ exchange on the brush border membranes (BBMs) of absorptive villus cells. However, the vast majority of animal models of IBD used to study IBD-associated diarrhea exhibit inflammation in the colon, with limited studies focused on electrolyte and fluid transport defects in Crohn’s-like ileitis, most commonly occurring in the terminal ileum [1]. We have extensively used the rabbit model of chronic enteritis to investigate impaired nutrient transport in chronic small intestinal inflammation [11,12,13,14,15] and also demonstrated glucocorticoid reversal of the inhibition of Cl^−^/HCO_3_^−^ exchange [16] and nitric oxide-mediated modulation of NaCl absorption [17].

In IBD, altered absorption of NaCl and water causing diarrhea could be mediated by inflammatory mediators known to be elevated in the inflamed intestine. Immune-inflammatory mediators such as the metabolites of arachidonic acid (AA) are known to infiltrate into the mucosa during intestinal inflammation. We and others have shown these metabolites to be increased in IBD patients and animal models of IBD [11,18,19]. Cytosolic phospholipase A2 (PLA2) catalyzes the release of AA from membrane phospholipids. In turn, the AA metabolites (AAMs) formed via cyclooxygenase (COX) or lipoxygenase (LOX) pathways may contribute to clinical diarrhea in IBD. Prostaglandins (PGs) and leukotrienes (LTs), the two major groups of AAM formed via the COX and the LOX pathways, respectively, have been implicated in the pathogenesis of a number of inflammatory diseases, including IBD [19]. We have previously shown in a rabbit model of chronic ileitis that prostaglandins, but not leukotrienes, alter NaCl absorption via inhibition of Cl^−^/HCO_3_^−^ exchange in ileal villus cell BBM, but without having any effect on Na^+^/H^+^ exchange [20]. In the current study, we used SAMP1/YitFc (SAMP1) mice, which are models of spontaneous ileitis closely resembling human IBD, with AKR/J (AKR) as control to investigate the effects of chronic inflammation on NaCl absorption. Our results demonstrate significant inhibition of Cl^−^/HCO_3_^−^ exchange in the BBM of ileal villus cells of SAMP1 mice compared to AKR/J mice. Further, inhibition of the formation of AAM in general, and the COX pathway AAM in particular, have reversed the inhibition of Cl^−^/HCO_3_^−^ exchange in SAMP1 mice. 

## 2. Results

### 2.1. Histologic Illustrations of Chronically Inflamed SAMP1 Mice and AKR Control Mice Ileum

**Figure 1:**
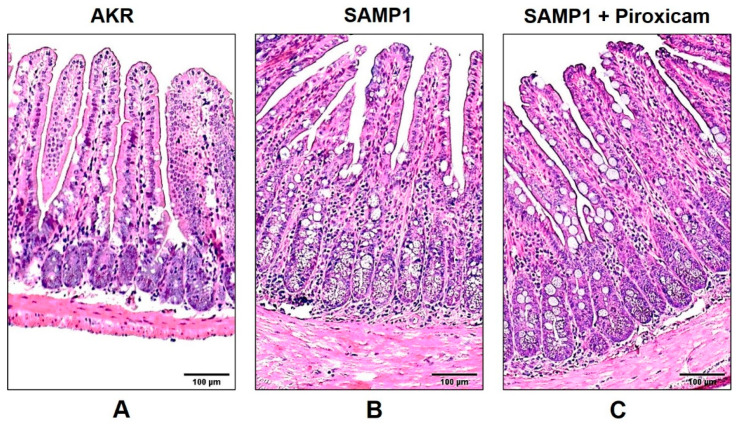
Illustrative photomicrographs of cross sections of the ileum of AKR and SAMP1 mice stained with hematoxylin and eosin (H&E). (**A**) The AKR mouse ileum depicts the typical long villi, short crypts and minimal intraepithelial immunocytes. (**B**) SAMP1 mouse intestine demonstrates crypt hypertrophy, villus blunting, and increased intraepithelial lymphocytes characteristic of inflammatory bowel disease (IBD). (**C**) In vivo treatment of SAMP1 mice with piroxicam, a COX pathway inhibitor, did not show any changes in the ileal structure in SAMP1 mice. Original magnification 10×. Scale bar 100 μm.

Figure 1 shows the ileal cross sections from 10-week-old SAMP1 and AKR control mice stained with hematoxylin and eosin (H&E; magnification ×10) staining. A normal structure in control AKR mice is shown as the typical long villi, short crypts and minimal intraepithelial immunocytes (Figure 1A). SAMP1 mice developed spontaneous, transmural inflammation of the terminal ileum characterized by discontinuous inflammatory infiltrates, crypt hypertrophy, villus blunting and distortion, and bowel wall thickening, resembling human IBD (Figure 1B). In vivo treatment of SAMP1 mice with piroxicam, a COX pathway inhibitor, did not show any morphological changes in SAMP1 mice, as shown in Figure 1C. Histologic results indicate that SAMP1 mice spontaneously develop lesions in their terminal ilea, reminiscent of human IBD, and in vivo treatment of SAMP1 mice with a COX pathway inhibitor has no effects on ileal structure. 

### 2.2. Inhibition of Cl^−^/HCO_3_^−^ Exchange Activity in BBMV Prepared from SAMP1 Mice Ileum

**Figure 2:**
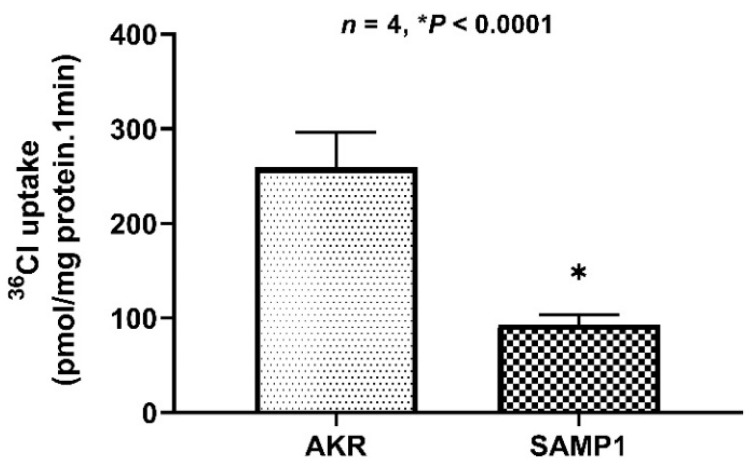
Inhibition of Cl^−^/HCO_3_^−^ exchange activity in villus cell BBMV of SAMP1 mice. Cl^−^/HCO_3_^−^ exchange activity (^36^Cl uptake, pmol/mg protein/min) was significantly inhibited in BBMV prepared from ileal villus cells of SAMP1 mice compared to AKR controls. *N* = 4, * *p* < 0.0001.

We have previously shown that Na^+^/H^+^ exchange is not altered in SAMP1 mice [21]. Since coupled NaCl absorption in the mammalian small intestine has been shown to be mediated by the dual operation of Na^+^/H^+^ and Cl^−^/HCO_3_^−^, in this study, we studied Cl^−^/HCO_3_^−^ exchange in villus cell BBMV of SAMP1 versus AKR mice. Cl^−^/HCO_3_^−^ exchange activity, defined as HCO_3_^−^-dependent and DIDS-sensitive ^36^Cl uptake, was present in the BBM of ileal villus cells from both AKR and SAMP1 mice. However, the results presented in Figure 2 show that Cl^−^/HCO_3_^−^ exchange is significantly inhibited in SAMP1 BBMV compared to the AKR controls. 

### 2.3. Role of Arachidonic Acid Metabolites (AAMs) in Inhibiting Cl^−^/HCO_3_^−^ Exchange in SAMP1 Mice

**Figure 3:**
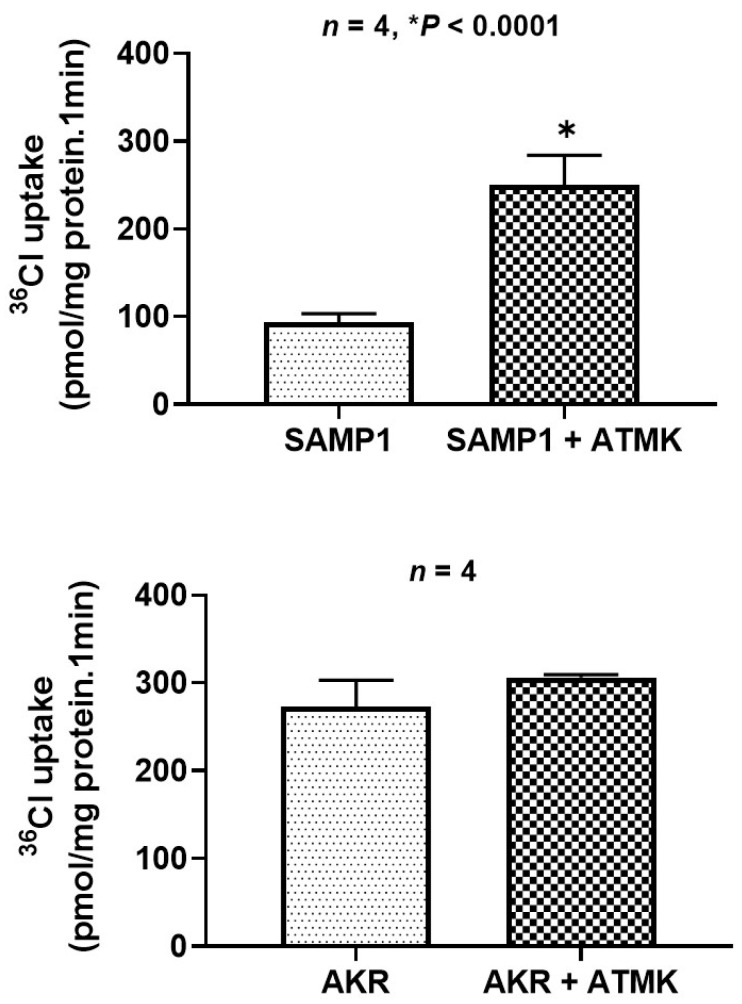
ATMK treatment of SAMP1 mice reversed the inhibition of Cl^−^/HCO_3_^−^ exchange activity. Mice were treated intraperitoneally with ATMK (3 mg/kg), inhibitor of AAM formation, for 2 days. Cl^−^/HCO_3_^−^ exchange activity was measured in ileal villus cell BBMV. ATMK treatment stimulated Cl^−^/HCO_3_^−^ exchange in SAMP1 mice compared to untreated mice (upper panel) *n* = 4, * *p* < 0.0001; in AKR mice, ATMK had no effect on Cl^−^/HCO_3_^−^ exchange (lower panel).

Soluble mediators generated in response to immune activation play important roles in mucosal inflammation in IBD [19]. The AAMs, including prostaglandins, comprise an important group of soluble mediators elevated in IBD and exert multiple effects [19]. AAMs have been shown to stimulate Cl^−^ secretion [22]; however, their effects on Cl^−^ absorption are not known. Therefore, we used Arachidonyl Trifluoro Methylketone (ATMK), an inhibitor of AAM formation, to treat SAMP1 and AKR mice intraperitoneally (3 mg/kg) for 2 days. Next, Cl^−^/HCO_3_^−^ exchange activity was measured in ileal villus cell BBMV from SAMP1 and AKR mice. As shown in Figure 3, Cl^−^/HCO_3_^−^ exchange was significantly increased in response to ATMK treatment in SAMP1 mice (upper panel), whereas in AKR controls, ATMK showed no effect on Cl^−^/HCO_3_^−^ exchange (lower panel). These results suggest that inhibition of Cl^−^/HCO_3_^−^ exchange in SAMP1 mice could be mediated by AAM generated in the inflamed mucosa.

### 2.4. Cyclooxygenase (COX) but Not Lipoxygenase (LOX) Pathway Metabolites of AA Inhibit Cl^−^/HCO_3_^−^ Exchange in SAMP1 Mice

**Figure 4:**
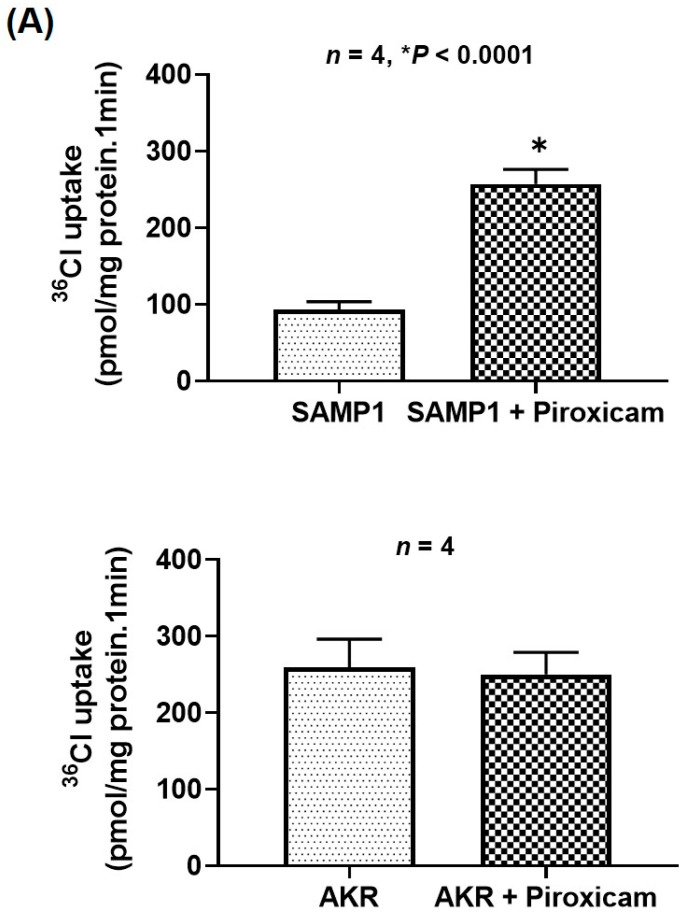

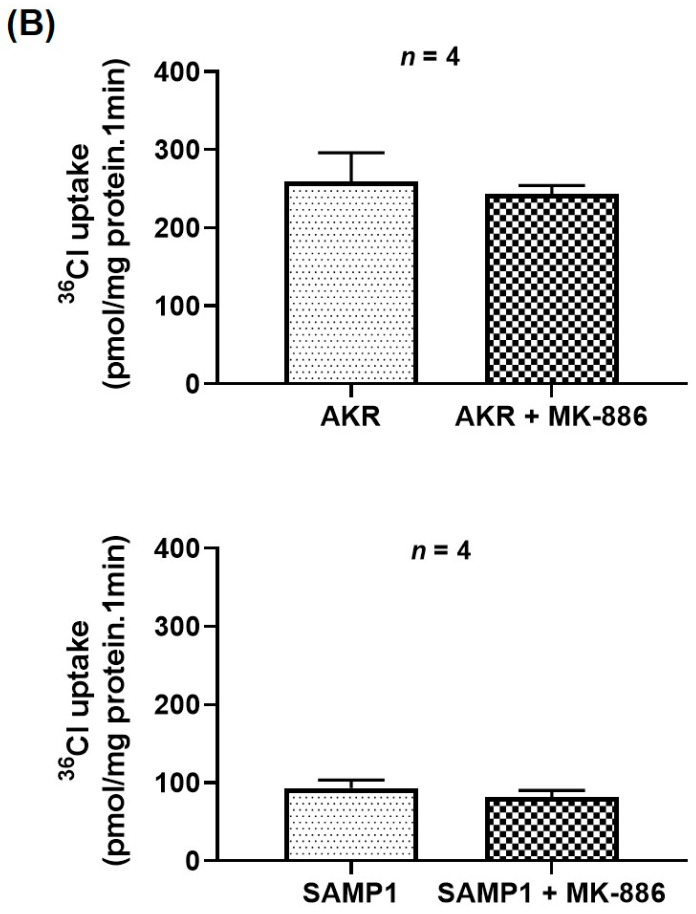
Effect of (**A**) piroxicam or (**B**) MK-886 treatment on Cl^−^/HCO_3_^−^ exchange activity in SAMP1 mice. Mice were treated intraperitoneally for 2 days with piroxicam (10 mg/kg) or MK-886 (0.5 mg/kg) to inhibit COX or LOX pathways of AAM formation, respectively, and then, Cl^−^/HCO_3_^−^ exchange activities were measured in BBMVs. (**A**) Piroxicam treatment significantly stimulated Cl^−^/HCO_3_^−^ exchange in SAMP1 mice (upper panel) (*n* = 4, * *p* < 0.0001), but had no effect in AKR mice (lower panel). (**B**) MK-886, while showing no effect on Cl^−^/HCO_3_^−^ exchange in AKR mice (upper panel), did not block the inhibition of Cl^−^/HCO_3_^−^ exchange in SAMP1 mice (lower panel).

Major pathways of AA metabolism relevant to IBD are induced via activation of COX (COX-1, COX-2) and LOX (5-LOX, 12-LOX). To investigate which AAM, COX or LOX members are involved in inhibiting Cl^−^/HCO_3_^−^ exchange, SAMP1 and AKR mice were treated intraperitoneally for 2 days with piroxicam (10 mg/kg) or MK-886 (0.5 mg/kg) to inhibit COX or LOX pathways of AAM formation, respectively, and then, Cl^−^/HCO_3_^−^ exchange activities were measured in BBMVs. Results presented in Figure 4 show that piroxicam treatment diminished the inhibition of Cl^−^/HCO_3_^−^ exchange in SAMP1 mice (4A, upper panel) but had no effect on Cl^−^/HCO_3_^−^ exchange in AKR mice (4A, lower panel). On the other hand, MK-886, while showing no effect on Cl^−^/HCO_3_^−^ exchange in AKR mice (4B, upper panel), did not block the inhibition of Cl^−^/HCO_3_^−^ exchange in SAMP1 mice (4B, lower panel). These results suggest that in SAMP1 mice, inhibition of Cl^−^/HCO_3_^−^ exchange is mediated by the COX pathway metabolites of AA, not by the LOX pathway AAMs.

### 2.5. Kinetic Analysis of AAM-Mediated Inhibition of Cl^−^/HCO_3_^−^ Exchange in SAMP1 Mice

**Figure 5:**
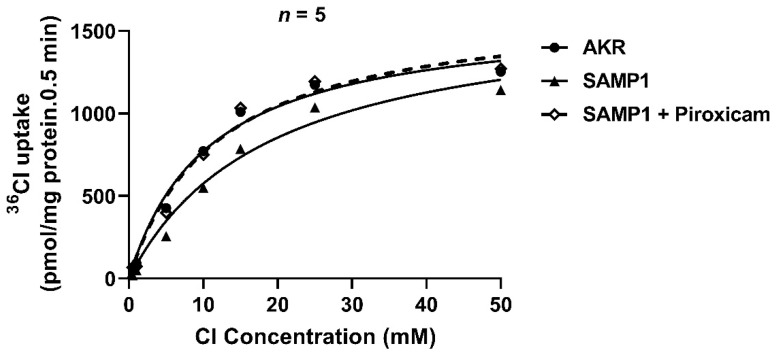
Kinetics of Cl^−^/HCO_3_^−^ exchange in villus cells from AKR, SAMP1, and piroxicam-treated SAMP1 mice. DIDS-sensitive ^36^Cl uptake in different groups is shown as a function of varying concentrations of extracellular Cl^−^. Analysis of the kinetic parameters (K_m_ and V_max_) by GraphPad Prism software showed no significant alteration of V_max_ in all the groups. In SAMP1 mice, there was a significant increase in K_m_ (decrease in 1/K_m_) (*n* = 5) compared to AKR mice. Piroxicam treatment restored the K_m_ to normal, comparable to that of AKR mice.

Kinetic studies were performed to determine the mechanism of inhibition of Cl^−^/HCO_3_^−^ exchange by AAM by measuring Cl^−^/HCO_3_^−^ exchange activity in ileal villus cells from SAMP1/AKR at increasing concentrations (0.5–50 mM) of the substrate (Cl^−^). In the villus cells from SAMP1 mice, the affinity of the Cl^−^/HCO_3_^−^ exchanger protein for Cl^−^ was decreased, as compared to AKR villus cells, as represented by an increase in K_m_. Treatment of SAMP1 mice with piroxicam reversed this effect, restoring the K_m_ value comparable to AKR control (Table 1, Figure 5). These results suggest that the mechanism of inhibition of Cl^−^/HCO_3_^−^ exchange in SAMP1 mice villus cells was secondary to a decrease in the affinity of the exchanger (1/K_m_) for Cl^−^, rather than a reduction in the exchanger number, as the maximal rate of uptake by the exchanger (V_max_) was not altered.

### 2.6. Protein Levels of the BBM Cl^−^/HCO_3_^−^ Exchangers Are Not Altered in SAMP1 Mice

**Figure 6:**
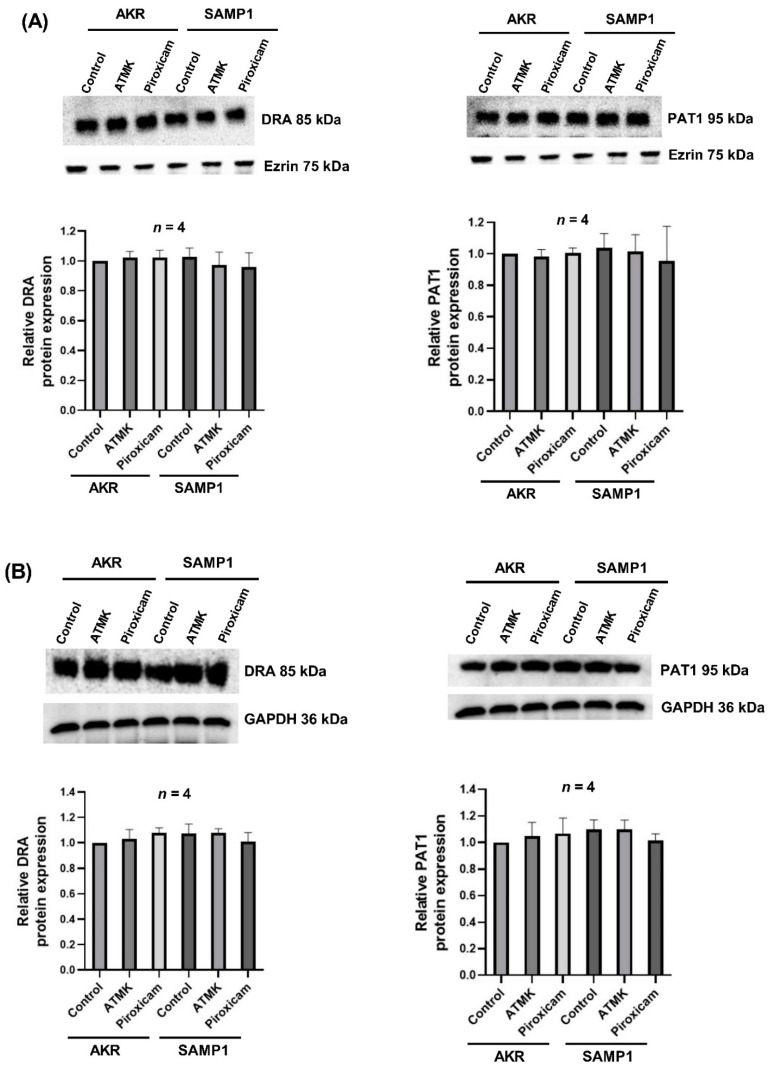
Protein levels of the BBM Cl^−^/HCO_3_^−^ exchangers (DRA and PAT1) are not altered in SAMP1 mice. Protein samples prepared from (**A**) BBM or (**B**) cellular homogenate protein preparations from AKR and SAMP1. SAMP1 treated with ATMK or piroxicam was loaded (50 µg/sample) and separated on a 10% SDS-polyacrylamide gel, transferred onto a PVDF membrane, and probed with anti-DRA or anti-PAT1 antibodies as described in the Materials and Methods. Ezrin and GAPDH were used as the internal control. In both (**A**,**B**), a representative blot of 4 independent experiments (upper panels) and densitometric analyses of band intensities (lower panels) are shown. There were no alterations in the levels of DRA or PAT1 proteins in all groups.

In the mammalian intestine, the two key BBM Cl^−^/HCO_3_^−^ exchanger proteins of the SLC26 family, Down-Regulated in Adenoma (DRA, SLC26A3) and putative anion transporter-1 (PAT1, SLC26A6), mediate Cl^−^ absorption. The kinetic studies (Table 1, Figure 5) suggested that inhibition of Cl^−^/HCO_3_^−^ exchange in SAMP1 mice villus cells was secondary to a decrease in the affinity of the exchanger (1/K_m_) for Cl^−^, rather than a reduction in the exchanger number. Therefore, protein levels of the exchangers DRA and PAT1 were measured in the lysates of intact villus cells and in the BBM for all groups of mice. As shown in Figure 6, protein levels of DRA and PAT1 in the BBM (6A) or whole cell lysates (6B) were not altered in any of the groups (AKR, SAMP1, ATMK, piroxicam), further supporting our kinetics data that inhibition of Cl^−^/HCO_3_^−^ exchange in SAMP1 mice was not secondary to decreased number of exchangers.

### 2.7. Immunostaining of Mucosal DRA and PAT1 in SAMP1 Small Intestine

**Figure 7:**
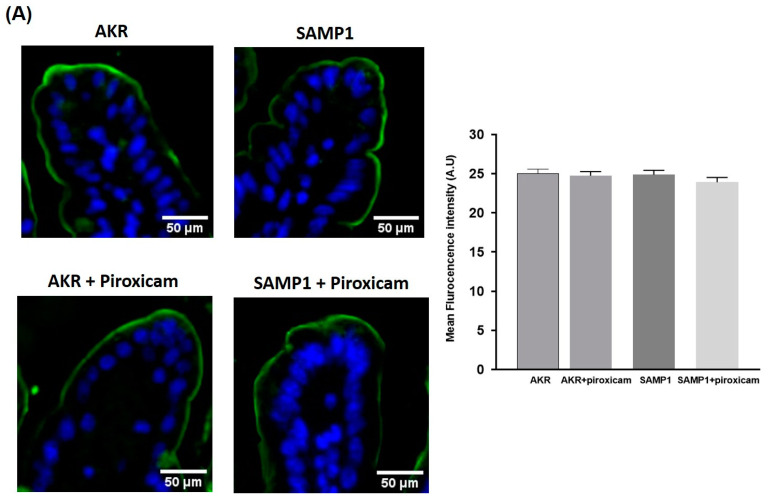

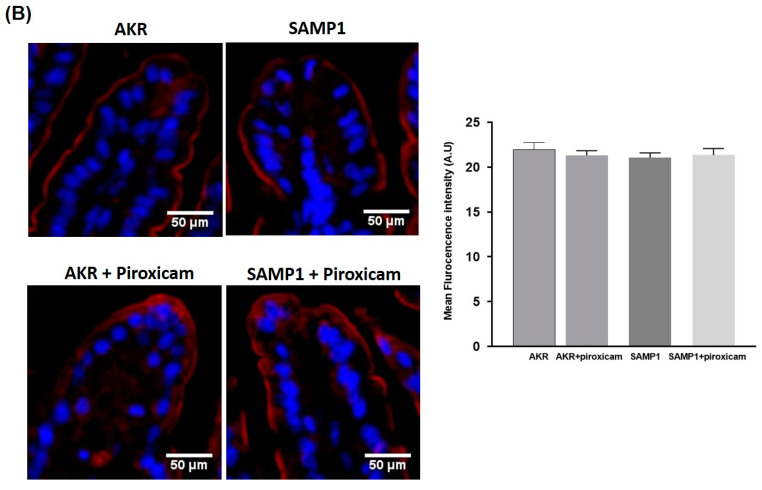
Immunofluorescence staining of DRA and PAT1 in small intestinal mucosa of SAMP1/AKR mice. (**A**) (Left panel): Immunofluorescence staining of small intestinal mucosal sections of AKR and SAMP1 mice for DRA (green) and DAPI (blue). Representative image of 4 independent experiments is shown. Images were captured at 20× magnification. (Right panel): Quantification of DRA immunostaining intensity by Image J software showed no alterations of DRA levels in AKR versus SAMP1 mice or in response to piroxicam treatments. (**B**) (Left panel): Immunofluorescence staining of small intestinal mucosal sections of AKR and SAMP1 mice for PAT1 (red) and DAPI (blue). Representative image of 4 independent experiments is shown. (Right panel): Quantification of DRA immunostaining intensity by Image J software showed no alterations of PAT1 protein levels in AKR versus SAMP1 mice or in response to piroxicam treatments.

To further confirm the mechanism of inhibition of Cl^−^/HCO_3_^−^ exchangers (DRA, PAT1) in SAMP1 mice, immunofluorescence studies were performed. Images were captured at 20× magnification. The levels of DRA protein in the BBM of villus cells was similar in AKR and SAMP1 mice and piroxicam treatment had no effect on DRA in either AKR or SAMP1 mice (Figure 7A). Similarly, levels of PAT1 protein in the BBM of villus cells were unaltered in AKR and SAMP1 mice or in response to piroxicam treatment (Figure 7B). The combined results of kinetic studies, Western blot and immunohistochemical studies clearly suggest that the inhibition of Cl^−^/HCO_3_^−^ exchange, mediated by DRA and/or PAT1, is secondary to altered affinity of the exchanger for Cl^−^.

## 3. Discussion

In the current study, we used SAMP1/YitFc (SAMP1) mice, a model of spontaneous ileitis closely resembling human inflammatory bowel disease (IBD), to investigate the impact of chronic inflammation on NaCl absorption. Our results demonstrated significant inhibition of Cl^−^/HCO_3_^−^ exchange, a component of coupled NaCl absorption in the brush border membrane (BBM) of intestinal villus cells in chronic inflammation. Chronic, recurring, and bloody diarrhea is a common and disabling morbidity of inflammatory bowel disease (IBD), a debilitating inflammatory disease of the intestine. Dysregulated fluid and ion transport is a hallmark of most diarrheal diseases [23,24]. In IBD, activation of the immune system results in the production of soluble mediators by the immune cells, causing mucosal injury and chronic inflammation. In the inflamed mucosa, normal fluid and electrolyte (NaCl) transport, absorption and/or secretion across the epithelium are disturbed. Extensive studies focused on the molecular pathophysiology of IBD-associated diarrhea, however, suggest that impaired NaCl absorption, rather than anion secretion, primarily contributes to diarrhea in IBD [10]. The major route of NaCl absorption in the intestine is via a coupled electroneutral mechanism of Na^+^/H^+^ exchange and Cl^−^/HCO_3_^−^ exchange mediated by Na^+^/H^+^ exchanger 3 (NHE3, SLC9A3) and Down-Regulated in Adenoma (DRA, SLC26A3), respectively [25,26]. NHE2 and SLC26A6 (PAT1) expressed on the apical surface also mediate Na^+^/H^+^ and Cl^−^/HCO_3_^−^ exchange, respectively [25,26]. In a rabbit model of chronic intestinal inflammation, in many aspects resembling human IBD, we have previously shown unique mechanisms of alteration of nutrient absorption [11,12,13,14,15]. Indeed, in the same model, we have also shown glucocorticoid-mediated reversal of the inhibition of the inhibition of Cl^−^/HCO_3_^−^ exchange [16] and nitric oxide-mediated modulation of NaCl absorption [17]. 

Most of the studies reported so far on impaired NaCl absorption in IBD utilized animal models of acute or chronic inflammation of the colon, with limited studies performed in models of small intestinal inflammation that manifest Crohn’s disease, most commonly occurring in the terminal ileum [1]. In the current study, we used SAMP1/YitFc mice, a model of spontaneous ileitis closely resembling small intestinal inflammation in human IBD, to investigate the mechanisms of alteration of NaCl absorption. SAMP1/YitFc inbred mouse strain, which develops ileitis spontaneously, without chemical, genetic, or immunologic manipulation, is highly relevant to Crohn’s-like ileitis [1]. In this model, for the first time, we previously reported the role of inducible nitric oxide (iNO) in inhibiting Cl^−^/HCO_3_^−^ exchange in the brush border membrane (BBM) of ileal villus cells with no alteration of Na^+^/H^+^ exchange [27]. In the current study, we have investigated the mechanism of inhibition of Cl^−^/HCO_3_^−^ exchange and demonstrated the role of arachidonic acid metabolites (AAMs), known to be elevated in the inflamed mucosa, in mediating inhibition of Cl^−^/HCO_3_^−^ exchange. Akin to our previous report [27], there was significant inhibition of Cl^−^/HCO_3_^−^ exchange in villus cells and BBMV prepared from villus cells of SMAP1 mice as compared to the AKR controls. When these mice were treated with ATMK to inhibit AAM formation, inhibition of Cl^−^/HCO_3_^−^ exchange in SAMP1 mice was abrogated, with no effect in AKR mice. 

In most cells, arachidonic acid (AA) is present primarily as a component of plasma membrane phospholipids. Activation of phospholipases, particularly phospholipase A2 (PLA2), by various cellular or extracellular factors releases AA from phospholipids. Major pathways of AA metabolism relevant to IBD include cyclooxygenase (COX) and lipoxygenase (LOX) [19]. Therefore, we used inhibitors of COX and LOX pathways to treat SAMP1/AKR mice to ascertain which pathway AAM could inhibit Cl^−^/HCO_3_^−^ exchange in chronic inflammation. Our results showed that inhibition of COX pathway, but not LOX pathway of AAM formation, reversed the inhibition of Cl^−^/HCO_3_^−^ exchange in SAMP1 mice. These inhibitors, as expected, had no effects on Cl^−^/HCO_3_^−^ exchange in AKR mice. These results clearly suggest the role of prostaglandins, key AAMs formed via the COX pathway, in inhibiting Cl^−^/HCO_3_^−^ exchange in SAMP1 mice. These findings are also consistent with our earlier demonstration that prostaglandins, but not leukotrienes (LOX pathway AAM) regulate Cl^−^/HCO_3_^−^ exchange in villus cells of chronically inflamed rabbit ileum, further supporting the impact of elevated AAM, more specifically of the prostaglandins, on NaCl absorption in IBD [20]. Our kinetic studies further showed that AAM-mediated inhibition of Cl^−^/HCO_3_^−^ exchange in SAMP1 mice is secondary to the reduction in the affinity of the exchanger protein for Cl^−^, not due to an altered exchanger number. That there was no alteration of exchanger number was further addressed by measuring the total cellular and BBM levels of DRA and PAT1, the key Cl^−^/HCO_3_^−^ exchanger proteins in the mammalian intestinal BBM. Protein levels of DRA and PAT1 in the villus cells or in the purified BBM were similar in SAMP1 and AKR mice, which also did not change in response to any of the treatments.

Affinity of the SLC transporters for their respective substrates could be altered via post-translational modifications of the transporter proteins that commonly occur via addition of a functional group, such as phosphorylation and glycosylation [28]. Prostaglandins induce various signaling pathways via activation of their G-protein coupled receptors. In intestinal epithelial cells, EP2 and EP4, receptors for PGE2, a key prostaglandin formed from AA, are highly expressed [21]. Both EP2 and EP4 are coupled to Gαs proteins; therefore, upon activation, increase intracellular cyclic AMP (cAMP) via activation of adenylate cyclase and in turn activate various downstream kinases. Therefore, future studies are needed to elucidate the role of phosphorylation and/or other likely post-translational modification of DRA and/or PAT1 in AAM-mediated inhibition of Cl^−^/HCO_3_^−^ exchange in SAMP1 mice.

In the current study, as well as in our previous studies in a rabbit model of ileitis [20], Cl^−^/HCO_3_^−^ exchange in the villus cell BBM was found to be inhibited by prostaglandins, but there was no effect on the Na^+^/H^+^ exchange, a process known to be commonly coupled to Cl^−^/HCO_3_^−^ exchange in the mammalian intestine. In addition to this major pathway of electroneutral NaCl absorption, there are pathways of nutrient-dependent absorption of Na^+^, mediated by Na^+^-glucose/amino acid symporters in the villus cell BBM [3]. In this regard, Na^+^/glucose cotransport mediated by BBM SGLT1 not only represents the unequivocal pathway for the absorption of the major nutrient glucose, but also plays a critical role in maintaining overall fluid and electrolyte homeostasis [29]. Indeed, Na^+^-glucose co-transport is the basis of Oral Rehydration Solution (ORS), used to treat dehydration due to diarrhea [30,31,32]. It seems quite logical to speculate that in IBD Na^+^/glucose cotransport and/or other Na^+^/nutrient symport mechanisms could have greater relevance to account for impaired NaCl absorption, which is further supported by the fact that the absorption of both electrolytes and nutrients (such as glucose) is substantially inhibited in this chronic inflammatory disease [33]. Therefore, it will be important in future studies to investigate the impact of elevated levels of AAM on Na^+^/glucose cotransport and/or other Na^+^/nutrient symport processes in the pathophysiology of IBD-associated diarrhea. 

In summary, the current study demonstrates that metabolites of arachidonic acid generated via COX pathway inhibit Cl^−^/HCO_3_^−^ exchange by decreasing the affinity of the exchanger protein for Cl^−^. Since prostaglandins, COX pathway AAMs, are known to be elevated in IBD, modulation of NaCl absorption by prostaglandins appears to be a significant factor contributing to IBD-associated diarrhea.

## 4. Materials and Methods

### 4.1. Chemicals and Antibodies

Arachidonyl Trifluoro Methylketone (ATMK), piroxicam and MK-886 were purchased from Cayman Chemical Company (Ann Arbor, MI, USA). Anti-DRA antibody raised in goat (sc-34939) and anti-PAT1 antibody raised in goat (sc-26728) were obtained from Santa Cruz Biotechnology (Dallas, TX, USA).

### 4.2. Mouse Model of Chronic Ileitis

Male SAMP1/YitFc (SAMP1) and age matched control AKR/J (AKR) male mice obtained from Jackson Laboratory (Farmington, CT, USA) were maintained in a 12 h light/dark cycle with free access to food and water in the Animal Care Facility at Byrd Biotechnology Science Center, Marshall University. After a week of acclimatization, the animals were included in the study at 10 weeks of age. SAMP1 and AKR mice were treated intraperitoneally for 2 days with ATMK (3 mg/kg), or with piroxicam (10 mg/kg) or MK-886 (0.5 mg/kg) and were euthanized at the end of the second day of treatment with excess carbon dioxide exposure. All the animal studies described in the manuscript were carried out strictly in accordance with the procedural and ethical regulations of Marshall University’s Institutional Animal Care and Use Committee (IACUC approval number 743, Approval date 17th May 2020).

### 4.3. Isolation of Villus Cells

Villus cells from the scrapped small intestinal mucosa of the animals were isolated by a calcium chelation technique as previously described by us [34,35]. Briefly, the distal small intestine extracted from these mice were filled with cell isolation buffer (0.15 mM EDTA, 112 mM NaCl, 25 mM NaHCO_3_, 2.4 mM K_2_HPO_4_, 0.4 mM KH_2_PO_4_, 2. mM l-glutamine, 0.5 mM β-hydroxybutyrate, and 0.5 mM dithiothreitol; gassed with 95% O_2_ and 5% CO_2_; pH 7.4) and incubated in 37 °C water bath for 3 min, followed by gentle palpitation for another 3 min to facilitate cell separation. The isolation buffer with the isolated villus cells was then drained from the intestine and centrifuged at 100× *g* for 3 min. The cell pellet thus obtained was flash frozen immediately in liquid nitrogen and stored at −80 °C until experimental use. 

### 4.4. Preparation of Brush Border Membrane Vesicles (BBMVs)

Ileal villus BBM vesicles (BBMVs) from mice were prepared by Mg^++^ precipitation and differential centrifugation [36,37].

### 4.5. Measurement of Cl^−^/HCO_3_^−^ Exchange Activity

Cl^−^/HCO_3_^−^ exchange activity was measured in BBMVs by rapid-filtration technique as previously described [17,20]. BBMVs were suspended in vesicle medium and either 50 mM KHCO_3_ (vesicle gassed with 5% CO_2_ and 95% N_2_) or 50 mM potassium gluconate (vesicle gassed with 100% N_2_). The reaction was started by adding 5 μL of vesicle to 95 μL reaction mixture with or without 1 mM 4,4-diisothiocyanatostilbene-2,2-disulfonic acid disodium salt (DIDS), a potent anion exchange inhibitor [17,20]. The uptake was stopped at 60 s with ice-cold stop solution containing 50 mM HEPES-Tris buffer (pH 7.5), 0.10 mM MgSO_4_, 50 mM potassium gluconate and 100 mM N-methyl-d-glucamine (NMG) gluconate [17,20]. Each reaction mixture was then filtered on a 0.45 μm Millipore (HAWP) filter and washed twice with 5 mL of the ice-cold stop solution. The filter was then dissolved in 5 mL scintillation fluid (Ecoscint, National Diagnostics), and radioactivity was determined in a Perkin Elmer Tri-Carb LSC 4910TR Scintillation Counter. The uptake numbers were calculated as HCO_3_-dependent DIDS-sensitive ^36^Cl^−^ uptake to determine Cl^−^/HCO_3_^−^ exchange activity.

### 4.6. Kinetic Studies

To determine the kinetic parameters, Cl^−^/HCO_3_^−^ exchange activity was measured in intact villus cells isolated from AKR mice and SAMP1 mice treated or untreated with ATMK for 2 days. The isolated cells (100 mg) were suspended in buffers containing 5 mM NMG gluconate and 50 mM HEPES-Tris (pH 7.5) with 100 mM KHCO_3_ or 100 mM K-gluconate. The uptake reaction was started by adding the suspended cells to a reaction mixture containing 5 mM NMG ^36^Cl^−^, 150 mM potassium gluconate, 50 mM MES-Tris (pH 5.5) and varying concentrations of HCl (0.5, 1, 5, 15, 25 and 50 mM) with or without 1 mM DIDS. The uptake was stopped at 30 s with the addition of an ice-cold stop solution containing 50 mM HEPES-Tris buffer (pH 7.5), 0.10 mM MgSO_4_, 50 mM potassium gluconate and 100 mM NMG gluconate. Each reaction mixture was then filtered on a 0.65 μm Millipore (DAWP) filter and washed twice with 5 mL of the ice-cold stop solution. The filter was then dissolved in 5 mL scintillation fluid (Ecoscint, National Diagnostics) and radioactivity was determined as described above. Uptake numbers that were obtained from these experiments were analyzed with GraphPad Prism 8 (GraphPad Software Inc., San Diego, CA, USA) for Michaelis–Menten kinetics using a nonlinear regression data analysis to derive kinetic parameters V_max_ and K_m_.

### 4.7. Western Blot

Homogenates of isolated villus cells were prepared by first centrifuging cells at 8000× *g* at 4 °C for 5 min and solubilizing the resulting pellet in radioimmunoprecipitation (RIPA) buffer containing protease inhibitors (Santa Cruz). Cellular extracts in RIPA were then centrifuged at 8000× *g* at 4 °C for 5 min, and the supernatant was measured for protein content with a NanoDrop Spectrophotometer (Thermo Scientific, Waltham, MA, USA). Proteins from BBM preparations were solubilized and extracted in RIPA buffer as above. Proteins were separated on 8% polyacrylamide gel, transferred onto a polyvinylidene difluoride membrane, blocked in 5% BSA for 1 h as previously described [38] and incubated with primary anti-DRA or anti-PAT1 antibodies at 4 °C overnight. Horseradish peroxidase coupled specific secondary antibodies were used to bind to DRA and PAT1 bound primary antibodies before detecting chemiluminescence with ECL Detection Reagent (GE Healthcare, Chicago, IL, USA). The density of the DRA and PAT1 specific protein bands were quantitated with a densitometric scanner FluorChemTM instrument (Alpha Innotech, San Leandro, CA, USA) and normalized with ezrin (MAB3822-C, Millipore, Temecula, CA, USA) and GAPDH (MA5-15738, Thermo Fisher Scientific, Waltham, MA, USA) bands. 

### 4.8. Histology and Immunohistochemistry

A portion of the distal ileum was tissue was fixed in 10% (*v*/*v*) neutral-buffered formalin (Sigma Aldrich, St Louis, MO, USA) and embedded in paraffin. Sections (5 μm) from the formal fixed tissue were obtained with a microtome and were mounted on glass slides. Paraffin was removed from the sections by incubating the slides with xylene, and sections were hydrated gradually by incubating with graded ethanol. Sections were stained with hematoxylin and eosin (H&E; Sigma Aldrich, St Louis, MO, USA) staining. For the immunofluorescence study, antigen retrieval was performed by incubating the sections with 10 mM sodium citrate buffer, pH 6, at 95 °C for 10 min as described previously (20). After washing with PBST (0.05% Tween-20 in phosphate buffered saline), sections were incubated in blocking buffer (2% bovine serum albumin) for 1 h at room temperature. The sections were then incubated for 1 h at room temperature with antichicken DRA primary antibody (custom antibody services provided by Invitrogen Life Technologies), at 1:100 dilution and anti-PAT1 primary antibody (sc:515230, Santa Cruz Biotechnology, Inc. USA) at 1:100 dilution. Excess antibody was removed by washing with PBST and incubated with Alexa Fluor 488 goat antichicken (A11039; Invitrogen Molecular Probes, Carlsbad, California) or Alexa Fluor 594 goat antimouse (A11032; Invitrogen Molecular Probes, Carlsbad, California) for DRA and PAT1, respectively, at 1:500 dilution for 1 h at room temperature. Sections were then washed thrice in PBST, and the nucleus was stained with 4, 9, 6-diamidino-2-phenylindole (DAPI) mount, i.e., Fluoroshield mounting medium with DAPI (ab104139, Abcam, USA) and observed with EVOS FL Cell imaging system. Images were quantified by using Image J software.

### 4.9. Statistical Analyses

Results are shown as means ± standard error of mean (SEM). Statistical analysis (unpaired t test) was performed with GraphPad Prism 7 software to derive statistical significance between data derived from different experimental conditions. A *p* value of <0.05 was considered statistically significant. The *n* number for any set of experimental data refers to experiments performed with villus cells or BBMV preparations or protein extracts obtained from *n* different animals of each experimental group.

## Figures and Tables

**Table 1:** Kinetic parameters for Cl^−^/HCO_3_^−^ exchange. The affinity (1/K_m_) of Cl^−^/HCO_3_^−^ exchanger for substrate (Cl^−^) was significantly decreased in the villus cells from SAMP1 mice compared to the control. The decrease in affinity was completely restored to normal in the villus cells obtained from piroxicam-treated SAMP1 mice.

	V_max_ (nmol/mg Protein/min)	K_m_ (mM)
AKR	1.60 ± 0.02	10.81 ± 0.26
SAMP1	1.65 ± 0.05	18.84 ± 0.76
SAMP1 + Piroxicam	1.66 ± 0.04	11.83 ± 0.48

## Data Availability

Not applicable.
